# Biofuel production as a promising way to utilize microalgae biomass derived from wastewater: progress, technical barriers, and potential solutions

**DOI:** 10.3389/fbioe.2023.1250407

**Published:** 2023-08-16

**Authors:** Qilin Zheng, Ruoxu Ning, Meng Zhang, Xiangyuan Deng

**Affiliations:** ^1^ College of Biotechnology, Jiangsu University of Science and Technology, Zhenjiang, China; ^2^ Zhenjiang Zhongnong Biotechnology Co., Ltd., Zhenjiang, China; ^3^ Key Laboratory of Ecological Impacts of Hydraulic-Projects and Restoration of Aquatic Ecosystem of Ministry of Water Resources, Institute of Hydroecology, Ministry of Water Resources & Chinese Academy of Sciences, Wuhan, China

**Keywords:** biofuel production, microalgae biomass, wastewater, progress, technical barriers, potential solutions

## 1 Introduction

Relative to conventional techniques for wastewater treatment (e.g., activated sludge and trickling filters), microalgae-based wastewater treatment has many advantages, such as low energy demand and operational cost, high removal rate of pollutants, reduction of greenhouse gas and sludge formation, and recovery of nutrients in the form of algal biomass (Sharma et al., 2022). Thus, it has received extensive attention in recent years, and been recognized as a safety, promising, and efficient alternative replacing the conventional techniques (Shahid et al., 2020). According to the published literature, some microalgae species (e.g., *Chlorella* sp., *Scenedesmus* sp., *Nannochloropsis* sp., *Botryococcus* sp., *Coelastrum* sp., *Chlamydomonas* sp., and *Dunaliella salina*) are reported to be able to treat wastewater at lab-scale, pilot-scale, or large-scale (Zhou et al., 2014; Ahmad et al., 2022). But levels of removal capacity and biomass utilization remarkably depend on the characteristics of algal species and physicochemical properties of wastewaters (Zhou et al., 2014; Deng et al., 2020).

Although biomass production is one of these advantages, excessive heavy metals, organic pollutants, and some pathogens are found in the algal biomass. For example, nine drugs (i.e., oxytetracycline, enrofloxacin, danofloxacin, tiamulin, ciprofloxacin, sulfadiazine, sulfadimidine, tylosin, and progesterone) are detected in the algal biomass from photobioreactors fed with piggery wastewater (López-Serna et al., 2022). Moreover, different kinds of heavy metals (e.g., cadmium, hexavalent chromium, mercury, nickel, lead, arsenic, copper, and zinc), a Gram-negative pathogen (*Escherichia coli*), and three pharmaceuticals or personal care products (i.e., hydrocinnamic acid, caffeine, and bisphenol A) could be found in the algal biomass derived from wastewater (Álvarez-González et al., 2023). Presence of these contaminants in algal biomass makes it unable to become a high-quality raw material for the production of food, feed, fertilizers, cosmetics, pharmaceuticals, and nutraceuticals. Thus, how to exploit the algal biomass effectively is one of the main challenges facing the technique of microalgae-based wastewater treatment.

**FIGURE 1:**
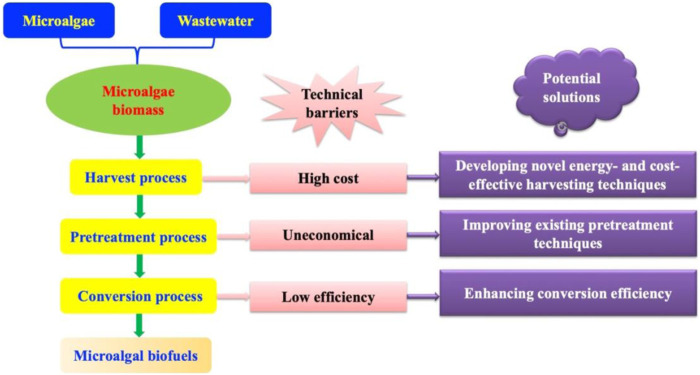
Technical barriers and potential solutions of biofuel production from microalgae biomass derived from wastewater.

Based on the published literature, biofuel production may be a very promising and practical solution to utilize microalgae biomass derived from wastewater (Deng et al., 2018b), but some key factors limiting its industrial application still persist, such as shortage of low-cost harvesting techniques, uneconomical pretreatment methods of microalgal biomass, and low efficiency of conversion process. In this paper, progress, technical barriers, and potential solutions in biofuel production from algal biomass cultivated in wastewater have been summarized (Figure 1). It is hoped that opinions listed in this paper could prevent the overly optimistic attitudes in this field, and spur researchers to find out practically-feasible solutions to the technical barriers.

## 2 Progress, technical barriers, and potential solutions

### 2.1 Shortage of low-cost and effective harvesting techniques

As we know, size of most microalgal cells is in the range of 2–50 μm, biomass concentration in large-scale cultivation ranges from 0.5 to 2 gL^-1^, and charge of algal cells is often negative (Min et al., 2022). These characteristics would lead to high cost of harvesting process, which is reported to be up to 20%–30% of the microalgal biomass cost or 50% of the total biofuel production cost (Muradov et al., 2015; Japar et al., 2017; Najjar and Abu-Shamleh, 2020). Thus, it is urgent to seek for simple and effective harvesting techniques, which could be used to harvest algal cells in low cost.

Nowadays, various harvesting techniques (i.e., centrifugation, sedimentation, flocculation, flotation, and filtration) have been tested to harvest algal cells cultivated in wastewater (Muradov et al., 2015; Eldiehy et al., 2022). Each of these techniques has its advantages and disadvantages. For instance, centrifugation is very efficient in concentrating microalgal cells with lower contamination possibility, but its cost is high (about 0.1 USD m^-3^ algal culture) (Najjar and Abu-Shamleh, 2020). Although cost of flocculation (about 0.01 USD m^-3^ algal culture) is significantly lower than that of centrifugation, it does not seem like a good harvesting technique because of its disadvantages, such as contamination to the harvested biomass (chemical flocculation), electrode material-dependence (physical flocculation), and high demand of bio-flocculants (bio-flocculation) (Vandamme et al., 2013; Eldiehy et al., 2022). Currently, low-cost and effective techniques have not been reported for harvesting microalgae biomass derived from wastewater. Although some emerging harvesting techniques, including flocculation using magnetic microparticles (Seo et al., 2015), flocculation using natural biopolymer (Taghavijeloudar et al., 2022), sedimentation using polymers (Yang et al., 2021), and magnetic membrane filtration (Zhao et al., 2020), have been proposed and carried out practically, these techniques also have their disadvantages, such as high cost and complicated operating steps. Thus, more novel energy- and cost-effective techniques should be further investigated to harvest microalgal biomass cultivated in wastewater.

### 2.2 Uneconomical pretreatment methods of microalgal biomass

Rigidity of cellular structure can influence extraction efficiency of biomolecules in microalgal biomass, and thus the biomass is necessary to be pretreated before being used to produce biofuels (Agarwalla et al., 2023). Recently, various pretreatment methods have been employed in biofuel production from microalgal biomass, such as physical pretreatment (e.g., bead milling, extrusion, microwave, ultrasound, and pulse electric field), chemical pretreatment (e.g., acid hydrolysis, alkaline hydrolysis, deep eutectic solvents, and ionic liquids), and physicochemical pretreatment (e.g., hydrothermal, supercritical fluids extraction, pressurized liquid extraction, and hydrothermal carbonization) (Agarwalla et al., 2023). Disadvantages of these methods are energy-intensive, high cost, and use of hazardous chemicals (Bhushan et al., 2023). For example, 6.00 and 0.23 kWh would be consumed when high-pressure homogenization and sonication are used to pretreat 1 kg algal biomass, respectively (de Carvalho et al., 2020). Surfactant coupled ultrasonic pretreatment and nanoparticle-induced bacterial pretreatment incurs a biofuel production cost of 34.92 and 413.14 USD/t of microalgal biomass, respectively (Kavitha et al., 2023). Therefore, future investigations should focus on improvement of existing pretreatment techniques and development of novel pretreatment methods for decrement in cost and energy requirement of pretreatment in depth.

### 2.3 Low efficiency of conversion process

After pretreatment, biomolecules in microalgal biomass will be converted into different types of biofuels, which depend upon the biochemical compositions of biomass and technology type (Aliyu et al., 2021). Based on the published literature, traditional conversion methods are transesterification for biodiesel production, anaerobic digestion for bio-methane production, gasification and pyrolysis for syngas production, and pyrolysis, ultrasound/microwave-enhanced conversion, and hydrothermal pretreatment for bio-oil and bio-char production (Ebhodaghe et al., 2022). However, conversion efficiencies of these methods are not very high. For instance, conversion efficiencies range from 20% to 50% when *Scenedesmus obliquus* and *Phaeodactylum tricornutum* are anaerobically digested in a hybrid flow-through reactor at either mesophilic or thermophilic conditions for bio-methane production (Zamalloa et al., 2012). Conversion efficiencies are in the range of 55.5%–78.2% when bio-oil extracted from biomass of *Dunaliella tertiolecta* is used to produce biodiesel in a transesterification reactor, where mixture of sodium hydroxide and alcohol is selected as a catalyst (Tizvir et al., 2023). Therefore, conversion technologies and efficiencies need to be improved in the future for achieving higher conversion and meeting the economic viability concurrently.

## 3 Summary and recommendations

In order to meet the challenges in utilization of microalgae biomass derived from wastewater, biofuel production has received a great deal of interest (Deng et al., 2018a; Deng et al., 2020). However, the development of microalgal biofuels faces a series of technical barriers according to our research experiences and literature reviews. Firstly, the currently used harvesting techniques are not efficient and economical, suggesting that more novel techniques with both energy efficient and cost-effective should be investigated in the future. Secondly, existing pretreatment methods are energy-intensive, high cost, and use of hazardous chemicals, indicating that these methods should be improved in the future. Finally, traditional conversion process does not have high efficiency, which should be optimized furtherly. Therefore, biofuel production using microalgal biomass derived from wastewater on commercial scale is still a long way to go due to the above technical barriers. This paper has recommended some potential solutions, which may help investigators to find future trends in this field.
